# Genito-Urinary and Syphilitic Diseases

**Published:** 1899-03

**Authors:** Byron H. Daggett

**Affiliations:** Buffalo, N. Y.


### GENITO-URINARY AND SYPHILITIC DISEASES.

CONDUCTED BY BYRON H. DAGGETT, M. D., Buffalo, N. Y.

### STAGES AND FORMS OF SYPHILIS.

ADOMI (*Canadian Practitioner*, July, 1898,) says: "It is remarkable how during all these centuries syphilis has remained sharply distinguished from all other forms of human disease. In other contagious and infectious maladies we recognise common principles governing their cause and effect; a common principle underlying the morbid changes in the tissues from the beginning to the end; a principle alike in the infant and the aged. Habitually the prognosis of syphilis is divided into three stages, as well as into two types, prenatal and postnatal. Wagner, more than thirty years ago, pointed out that all syphilitic lesions might be referred to the development of a specific neoplasm and preached the doctrine of the unicity of each lesion.

Nevins Hyde, in Morrow's system of genito-urinary diseases, ably advocates the doctrine of the unity of syphilitic manifestations.

It is not necessary to have any recognisable first stage or cutaneous chancre for syphilitic infection. In the female the absence of any superficial or recognisable first stage is especially noticeable; often the disease is manifest only in the second stage.

Do we find syphilis without a primary chancre? Do the spermatozoa of a syphilitic father bear syphilitic virus? Is a syphilitic father infected through a chancre? Would not syphilitic virus permeating the insegmented ovum destroy it? Fetal syphilis must originate after impregnation. Fetal and infantile syphilomata are not inherited; they are congenital.

Syphilitic parents doubtless beget offspring, who manifest a constitutional dyscrasia, but they do not present the progressive syphilitic lesions of true syphilomata. Inherited and congenital syphilitic lesions are two very different things.

A syphilitic father begets a syphilitic child, the virus passing through the immune system of the healthy mother and is carried by the blood to the susceptible tissue of the child. The second stage of acquired syphilis is the first stage of the congenital disease.

Kopasi says "that it is possible to have a true specific indurated chancre not followed by any secondary effects." Finger says "it is well established that women who have borne syphilitised children and have themselves never shown any primary or secondary manifestations, many years afterward, present unmistakable tertiary lesions."

Up to this point, therefore, it may be laid down: (1) That from analogy, as from clinical history and absence of any indications of the same, in sundry cases there may be an absence of the primary cutaneous or epithelial manifestations of syphilis.

(2) That individuals may fail to present either primary or secondary symptoms that are recognisable, and yet eventually develop definite tertiary lesions of the disease.

(3) That where the subject is relatively insusceptible it is possible that the disease may be limited to the primary cutaneous manifestation not followed by secondary lesions.

(4) That, as with tuberculosis so with syphilis, the congenital form of the disease begins at what may be termed the secondary stage of the acquired disease, *i. e.*, the stage of general dissemination of the virus through the organism.

